# Racial Differences in Perceptions of Genetic Wellness Programs

**DOI:** 10.1177/08901171231184360

**Published:** 2023-07-12

**Authors:** Forrest Briscoe, Ifeoma Ajunwa, Angel Bourgoin, James Maxwell

**Affiliations:** 1 Smeal College of Business, 8082 The Pennsylvania State University, University Park, PA, USA; 2 33739 University of North Carolina School of Law, Chapel Hill, NC, USA; 3 1871 JSI Research & Training Institute, Boston, MA, USA

**Keywords:** health policy, racial minority groups, workplace, qualitative research, workplace wellness programs, genetic wellness programs, diversity, equity and inclusion, health assessment

## Abstract

**Purpose:**

Genetic wellness programs (GWPs) are a highly innovative workforce wellness product. Recently marketed to U.S. employers by at least 16 vendors, GWPs take advantage of low-cost DNA sequencing to detect genetic risk factors for an increasing array of diseases. The purpose of this research is to understand perceptions, concerns, and barriers related to GWPs, among employees from Black, White, and Asian backgrounds and different income levels.

**Approach:**

Qualitative study with 3 focus groups (FGs).

**Setting:**

Employees of large high-technology companies (deemed likely early GWP adopters).

**Respondents:**

21 individuals recruited online through *User Interviews.*

**Method:**

FG guide developed via literature review and landscape analysis, and pre-tested. FGs led by a trained moderator and audio-recorded. Transcripts content analyzed for key themes.

**Results:**

Nearly all respondents saw potential benefits to GWP participation for themselves or their families. However, there were profound differences in perceptions of risks to GWP participation between Black and White/Asian respondents. These differences surfaced in three broad areas: privacy and discrimination risks; family impact risks; and feelings about the employer. Willingness to participate in a GWP also varied between Black employee respondents and White and Asian employee respondents (including low-income White employees). Only 27% of Black employees would participate in GWP, compared to 90% of the other employees.

**Conclusion:**

Most employees appear likely to support employer adoption of GWPs. However, Black employees report significant concerns regarding participation. Addressing these concerns through program design would benefit all employees, and could increase trust and uptake of GWPs.

## Purpose

Amidst widespread adoption and updating of workforce wellness programs, particularly among large employers,^1-3^ a new form is emerging. Genetic wellness programs (GWPs) are one of the most innovative workplace wellness products to be offered in recent decades. GWPs take advantage of new low-cost DNA sequencing and analysis technologies, to detect genetic risk factors for an increasing number of diseases and conditions.

In a typical GWP, employees who choose to participate have their genetic data generated and analyzed, and then they receive a personalized report generated from that data. Reports include personal information about future risks for developing diseases such as hereditary cancers and heart conditions. Reports may also include drug-metabolism information used to tailor medications, as well as personalized lifestyle recommendations. GWP participants are often encouraged to enroll in fitness, nutrition, or other programs. Some GWPs give employees access to genetic counselors to discuss their reports, while other encourage employees to discuss them with their existing health care providers.^4-7^

In some respects, GWPs appear similar to other types of workforce wellness programs. GWP proponents believe they can contribute toward workforce health promotion, helping to control employer and employee health care costs, and helping employers attract, retain, and engage talented employees who appreciate access to cutting-edge benefits. Relative to biometric screenings or other traditional health risk assessments, GWPs require little time or effort for participants, who can simply mail in a saliva sample and receive their report via the internet— similar to consumer genetic tests like 23andMe. After an employer adopts a GWP benefit, employees then opt-in to participate, sometimes with incentives for doing so. In addition, the employer typically covers part or all of the program costs for enrolled participants.^4,5^

In other respects, GWPs appear distinct from traditional workforce wellness programs. The technology that enables GWPs is relatively new, and the science used to generate genetic reports is complex and rapidly evolving. As a premium and high-technology employee benefit, GWPs may be more likely to be offered by large employers that need to recruit and retain professional workers in competitive labor markets (such as firms in the information technology, finance & insurance, life sciences, and health care industries).^4-6^ Self-insured employers, and organizations with health-conscious workforces, may also be more likely to consider offering a GWP.^5^ The design of GWPs varies widely, as they offer different types of information and additional services to participants, and they have different policies for handling and using participant data.^4-6^ They are also currently regulated in the U.S. under a shifting patchwork of federal and state laws.^8-10^

As more employers add GWP benefit offerings, research is needed to understand how employees perceive them and what factors will influence acceptance and participation. If GWPs turn out to play a valuable role in promoting workforce health, then it will be important to assess participation across different employee populations. For example, if racial or ethnic minorities or lower-income employees participate in GWPs at lower rates, this could contribute to additional disparities in health status across the workforce. However, little information on GWP participate rates is available; one unpublished Vanderbilt survey of 5 employers offering GWPs reported a 25% employee participation rate.^6^

Employees are likely to make decisions about GWP participation based on their perceptions of the benefits and risks—perceptions which may vary across different employee populations. We know of only one study to date reporting on employee perceptions of GWPs. While that study found that most employees would participate in a GWP if it provided confidentiality and privacy protections, the study’s conclusions were limited because all respondents were the employees of a single genomic testing organization, and the sample included few minority or low-income respondents.^11^ Accordingly, the purpose of this research is to begin to investigate perceptions of GWPs, and potential barriers to participation in them, among Black, White, and Asian employees, and employees of different income levels.

## Approach

This description of the study approach follows the Consolidated Criteria for Reporting Qualitative Research (COREQ) guidelines.

### Design, Setting and Samples

A qualitative study was conducted with 21 employees, recruited to three separate focus groups. In the remainder of this article, we use the term “respondent” to refer to the members of these focus group, rather than the term “participant,” such that in this article the term participation only refers to GWP participation.

We sought and obtained a purposive sample of current U.S.-based employees of large high-technology companies. These companies were deemed to be the most likely early GWP adopters, based on our initial research into GWP vendors and their employer customers.^4^ Individuals were recruited through the *User Interviews* platform*.* This platform allowed us to pre-screen candidates who expressed an initial interest in the study (based on a short summary of the topic and time and compensation information), using these criteria: employment status (currently employed) and specific industry (high-technology). Pre-screening yielded a pool of eligible candidates for the first focus group, from which 8 were selected for gender and racial balance (see below for details of subsequent focus groups). The *User Interviews* platform also provided a streamlined process for compensating individuals.

A focus group guide was developed using a literature review and landscape analysis identifying the key features of this new wellness product. Because GWPs are new, we incorporated presentation of basic educational information about GWPs into the focus group guide. The focus group guide was then revised based on pre-testing focus groups conducted with university students. The focus group guide is shown in the Appendix.

The final employee focus groups were guided by a trained facilitator (A. Borgoin, Ph.D.), who introduced herself, as well as a second trained observer, to respondents at the start of each focus group. Focus groups were all conducted and audio-recorded on the *Zoom* video conferencing platform. They lasted 75 to 90 minutes, for which respondents were each compensated $90. Field notes were made after each focus group, and audio recordings were later transcribed.

### Analysis and Adjustment

After completing the first focus group, the author team met to discuss emerging themes. One striking theme was a clear difference in the comments of the one Black respondent, compared with the general pattern of responses from the White and Asian respondents. This led us to design the second and third focus groups to include more racial diversity in them. Specifically, the second focus group was entirely composed of Black respondents, and the third focus group was composed of a mix of Black, White, and Asian respondents from lower income backgrounds (but still working for high-technology companies). The resulting set of three transcripts provided a window into overlapping and diverging perceptions among different racial groups.

The final transcripts were analyzed using the content analysis approach to identify key themes. Transcript coding was conducted by three of the study authors, during the months following completion of the focus groups. Emergent themes were identified for each separate focus group, and the study team then discussed those themes. Themes were then merged and integrated across the three focus groups, and representative quotes were selected to represent each theme. Special attention was paid to areas of overlap and divergence between Black and White respondents (two Asian respondents tended to have responses that were similar to those of the White respondents, so they were not separated in the analysis below).

## Results

### Respondent Characteristics

Table 1 provides demographic information on the 21 individual respondents who joined the focus groups. Approximately half (52.4%) identified as female, and half (47.6%) as male. Respondents identified their race/ethnicity as 42.9% Black (including two individuals who identified as Bi-Racial), 38.1% White, and 9.5% Asian. Respondents ranged from young workers (20-29) to older workers (60-69). 23.8% of respondents reported household incomes of less than $50,000, which we considered low income for purposes of this study. Most respondents reported working for medium-sized or large employers. Finally, by design, all respondents worked full-time for U.S. high-technology companies in the “Computer Software/SaaS” industry or “Information Technology and Services” industries, where early employer adoption of GWPs was deemed to be most common.

**Table 1.** Focus Group Respondent Demographics.^a^

	n	(%)
Gender		
Male	10	(47.6%)
Female	11	(52.4%)
Race/ethnicity		
Black	11	(47.6%)
Asian	2	(9.5%)
White	8	(38.1%)
Hispanic	0	(0%)
Age		
20-29	6	(28.6%)
30-39	5	(23.8%)
40-49	5	(23.8%)
50-59	4	(19.0%)
60-69	1	(4.8%)
Household income		
Less than $50,000	5	(23.8%)
$50,000 to $99,999	8	(38.1%)
$100,000 or more	8	(38.1%)
Employer size		
Less than 1000 employees	1	(4.8%)
1001 to 5000 employees	8	(38.1%)
5001 to 10 000 employees	4	(19.0%)
10 001 or more employees	9	(42.9%)

^a^All respondents were working full-time in the “Computer Software/SaaS” or “Information Technology and Services” industries, and were living and working in the United States.

### Willingness to Participate in a Genetic Wellness Program

After providing basic information about GWPs, we asked respondents whether they would participate in a GWP if it were offered by their current employer. Overall, if their employer were to offer a GWP, 57% of respondents said they would participate while 33% said they would not (10% were undecided).

However, this overall pattern masked a wide gap in willingness to participate between White/Asian respondents and Black respondents. Specifically, only 27% of Black respondents said they would participate, compared to 90% of the other respondents (who were all White and Asian). Table 2 provides a detailed break-down of responses by respondent race.

**Table 2.** Focus Group Respondents’ Willingness to Participate in a GWP, by Race.

	White	Asian	Black	Total
Yes	7	2	3	12
Maybe	1	0	1	2
No	0	0	7	7
	8	2	11	21
% yes	88%	100%	27%	

With respect to respondent income levels, we did not find discernible differences in willingness to participate. As shown in Table 3, out of 5 lower-income respondents (defined as household income below $50,000), 40% said they would participate. However, looked at by race, a large majority of White/Asian respondents were willing to participate at *all three* income levels, while in comparison only 1 Black respondent was willing to participate from each of the three income levels. While these numbers are too small to draw statistical conclusions, the pattern suggests that race may be a more consistent influence on willingness to participate compared with income level. Our detailed analysis of respondent comments, discussed below, also revealed consistent differences by race but no apparent pattern based on income levels.

**Table 3.** Focus Group Respondents’ Willingness to Participate in a GWP, by Income Level.

	Household Income Less Than $50,000	Household Income $50,000 to $99,999	Household Income $100,000 or More	Total
Yes	2	4	6	12
Maybe	0	0	2	2
No	3	4	0	7
	5	8	8	21
% yes	40%	50%	75%	

### Perceived Benefits of a Genetic Wellness Program

When asked about the perceived benefits of a GWP, respondents tended to discuss benefits related to the usefulness of information that could be learned from the genetic testing. Three broad themes emerged: preventing diseases, helping their families, and finding better prescription medications. There were no major differences in perceived benefits based on respondent race. In addition to the quotes included below, Table 4 provides additional illustrative quotes for each theme.

**Table 4.** Perceptions of Benefits.

Useful information for prevention	I thought of it as an opportunity to potentially prolong my life by understanding what I could potentially be experiencing over the next 40 years and what I can do now as an adult to try and ensure that we can lessen the severity of a lot of those health issues. [Black Male]
	My father passed away from a heart attack. My family has high blood pressure. So knowing certain things of what I can do to prevent or lessen the chance of me expiring from those kind of illnesses, being that some of them are hereditary, what changes I can make in my life in the forefront to possibly prevent them, if I can, it definitely will help. I see that useful information. It’s still scary though, but it’s good to know. [Black female]
	I particularly liked the heart disease and cancer as well as the family. I think it would give an individual an idea if they’re predestined to certain types of cancers or heart risks, which could potentially save their lives. [White female]
	I’d like to know things I can take to my primary care physician. If it’s free from my employer, I can take it to my primary care physician and say, “hey, here are some of the things that I’ve seen. What do you recommend?” [white male]
Useful information for family	I think more information is always better than no information. Some of us don’t have any of this information. So just being able to make a educated determination on any of that information that we receive, could be life changing for not only you, but your kids, your grandkids and your posterity. [White male]
	I would like to know if I potentially have the risk of sending something down to my kids or something like that. [White female]
Useful information for prescriptions	I think the genetic potential is great as far as being able to figure out what drugs you might be allergic to and everything else. [Black male]
	To me, the main benefit coming out of it would be the whole drug piece of it because so much of as we get older, getting prescriptions for things, and so much of that is trial and error. We’ll try this prescription and it doesn’t work ... If we can do DNA testing that will narrow that down and accelerate that, I think that’s a huge benefit. [Black male]

For ***preventing disease***, many respondents saw the potential to learn information about their personal genetic predisposition for heart disease, cancer and other diseases. One respondent noted this could be particularly useful because they lacked information about family medical history: “… I don’t know what kind of health issues they may have had, and then their parents as well. So I think I can agree that it would be very helpful to know those things.” [White Male]. In general, respondents reflected an understanding that such information could be acted on through their own behavioral changes, and by discussing the information with their physician.

For ***helping their family***, several respondents were excited about the potential for information to be usefully applied to improve the health of their children or other family members, and one respondent alluded to the value in family planning. Finally, for prescription medications, a few respondents discussed the value of learning which medications would be more effective for them. One respondent explained “so much of that is trial and error … If we can do DNA testing that will narrow that down and accelerate that, I think that’s a huge benefit.”

### Perceived Risks of a Genetic Wellness Program

When asked about perceived risks, respondents discussed several wide-ranging concerns. These can be grouped into: concerns about inaccurate information; concerns about misuse of information, related to a lack of trust; concerns about genetic discrimination; and concerns about misuse by law enforcement. For the first two themes, inaccurate information and misuse of information, there were additional specific concerns voiced by Black respondents that related to their racial identity. Table 5 provides illustrative quotes for each theme.

**Table 5.** Perceptions of Risks.

Inaccurate information (in general)	Is the testing that good? Would they find something out? Then, you’re worried, maybe you tell people related to you, and it’s totally incorrect. [White Male]
	Are they an accredited lab, because that’s important, or are you using Bubba’s lab down the street? [Black female]
	Is there the opportunity that they could make a mistake? There could be a cross contamination of my genetic information with something else. I mean, I have no idea how they do it. Are they guaranteeing that this is at a 100% accuracy? [Black male]
Inaccurate information (specifically for Black people)	There’s no context as to the quality of the data, the depth of it. For example, most of the DNA information that’s out there in these databases tends to be more European. [Black male]
	If you’re getting data for, as an Asian person, that you’re at risk of this, there’s not that much data there and you don’t know that. [Black male]
Misuse/Lack of trust (in general)	Loss of control over my data. So, where my data is going to land? Is it going to some pharmaceutical company who’s going to use that as a tool to market some unwanted medication to me? And put me on a medication which I would normally not take when it is not required? [Black female]
	The largest risk I had was associated with whether my data would still be mine. And this came primarily as a result of [one company] being purchased by [another company], and big pharma started purchasing genetics companies. [Black male]
	I think in terms of the risk is to just the sensitivity of the information and how it could possibly be monetized, marketed at different individuals, really just … purposes that are detrimental to our safety even, so really honing in on those. [Black male]
	I’m an engineer, and I know basically almost every company sells your data. When you accept the terms of service, there’s always there, “hey, I’m going to sell your data.” That’s how most companies make their money. [Black female]
Misuse/Lack of trust (specifically for Black people)	These are things that have historically happened in the medical community. I mean, if we take a look at Henrietta lacks, where she donated some cells or some cell samples were taken for diagnosis for her, and these cells went on to be used for other medical things. Now, granted none of this was nefarious in nature, but it was the fact that she did not give consent for her DNA samples to be used for that. And, there’s a lot of examples of that throughout medical history. And so, when people are expressing this concern of my medical data or my genetic data being used, it might not necessarily be for a negative or be for a profit, but it could be used. And, there’s always that concern of, “I did not give my consent for my medical information to be used for this purpose, or my medical data to be used for this purpose.” … I think that’s the biggest concern is we don’t know the scope of what our data would be used for, and that’s the scary part. And, it’s a valid fear and concern because it’s happened before with people. [Black female]
	something that just should be stated because it’s like the elephant in the room is that Black people historically have a distrust of organizations that for this kind of thing, for health type stuff. So that should probably be addressed at some level as far as, “can we trust you” just from this specific demographic’s uncomfortableness around it? [Black male]
Genetic discrimination by insurers	I’ll tell you, this in the context of a risk management insurance sort of environment … it feels a little off-putting. I know how hard it is for me - I can’t even think of getting private insurance, and I had very benign cancer, okay, not something that would be terribly risky. This sort of thing … could be really challenging for people. And, once it’s kind of in that insurance world, even if it’s part of your workplace, I mean, it kind of follows you everywhere. So, I think the challenge is how do you maintain privacy? [White male]
	It just feels like it’s a way that can make medical care unavailable if you have certain genetic traits. [White male]
Misuse by law enforcement	it’s almost funny about the law enforcement grabbing your information off of there, not even about me, but maybe from my family history because I’m connected to Mickey mouse on the other end. They’re seeing that connection, and they’re like, “we found Mickey mouse because of your data.” I don’t want to be responsible for that. So it just continues to raise more questions. [Black female]
	Going back to the distrust thing, of course, the database on the law enforcement side, that’s how they caught the Golden state killer is because the DNA from a relative led them to him. But that was on a third database. How do we know that data’s not going to be transferred over? [Black male]

Respondents were concerned about ***inaccurate information*** from this type of genetic testing. For example, one respondent said, “being a preventive care, if their findings are not accurate, and we get put onto some sort of medication which is not required at all, that can lead to something else undesirable, so that’s something I’m worried about.” Some respondents perceived that there were not strict rules about the laboratories doing GWP testing. A related concern was that the information provided could be misinterpreted, by themselves or people around them with whom they had shared the information.

Concerns about inaccurate information were stronger among Black respondents, some of whom recognized that testing accuracy depended on genetic databases that lacked diversity. One respondent also said this was essentially a concern for all non-White racial and ethnic groups, stating “There’s no context as to the quality of the data, the depth of it. For example, most of the DNA information that’s out there in these databases tends to be more European.” [Black Male].

Concerns about ***misuse of information*** included concerns about what the GWP itself would do with the respondent’s genetic information, including selling it to other organizations which might in turn use that information to target them with products or otherwise influence their behavior in ways they would not want. Several respondents made a connection between the potential for GWPs to misuse and/or sell their data, with the tendency of technology companies and pharmaceutical firms to sell people’s personal data. One respondent connected this concern to a broader right to control over other types of personally identifiable information: “just like a fingerprint scan … I think you should be able to determine whether or not it can be sold to whoever, or if it shouldn’t.”

Concerns about misuse of information were also stronger among Black respondents, some of whom pointed out that a lack of trust in how they would be treated by health organizations was rooted in the long history of Black communities and families being abused by medical researchers. One respondent discussed the specific experience of Henrietta Lacks’ family.

Concerns about ***genetic discrimination*** were discussed in terms of insurance companies obtaining information about their genetic risks and using that information to exclude them from insurance or make it more expensive. This would be illegal for a health insurer to do (under federal law), but it is allowed and common for life insurance, long-term care and other forms of insurance.

Concerns about ***misuse by law enforcement*** revolved around the idea that family members could be harmed by the way police used genetic data to find matches to crime scene DNA. This concern also combined with the other misuse concerns raised, as one respondent discussed genetic data being moving from one organization’s database to another location where it could be searched by law enforcement, and respondents drew parallels between the right to control their DNA data and the right to control their fingerprints. This concern was only mentioned by Black respondents in our study, and may be related to Black distrust of law enforcement due to historical racism and violence.

Another distinct type of risk was also raised by several Black respondents: a concern that information about possible future diseases would ***produce anxiety*** without proving to be useful. For example, one respondent said: “I don’t know if I necessarily want to know about anything health-wise that could potentially happen to me in the future. … just knowing something and it being on my mind and the future and just always thinking about it. I would rather just live my life.” [Black Female] This line of thinking led this sub-set of Black respondents to conclude that they would rather not participate in a GWP for this reason.

This concern over the production of anxiety from using a GWPs stemmed in part from the probabilistic nature of genetic testing information: “Are they saying, ‘we’re only 50% accurate’ like ‘Hey, you might have cancer in five years, or maybe not!” Well, we all know that maybe *anything,* maybe. Give me something that gives me more surety that we are 90% accurate …. You’re going to get people in fear. Like, [she] was talking about that, ‘hey, you told me I might have cancer tomorrow,’ and now she’s freaked out every day. She’s actually hurting herself because she’s scared to death. So that can impact your health. Fear impacts your health, whether you know it or not.” [Black Female]

While this reflection on the relationship between probabilistic information, fear and anxiety, and health was shared among a subset of Black respondents, it is also worth noting that it was also mentioned by one White respondent, at least in general terms: “Sometimes it’s better to not know. The old saying ‘ignorance is bliss’ is very true.” [White Female]

Finally, a different stance on the risks of GWP participation was voiced by one respondent who believed that GWPs would naturally safeguard employee data from misuse since it was in the GWPs’ long-term interests to maintain employee trust: “if there’s any chance my data would be leveraged for nefarious purposes, I wouldn’t use that company or service. But, it’s very doubtful that would be the case, because that’s what the entire business or model is built on, right, consumer trust on not leaking out your genetic info and data... I think some of the fears are overblown in my opinion.” [White Male] However, this perspective did not receive obvious support from other respondents in their focus group.

### Additional Themes

When respondents were then asked how they would react if their employer included a ***financial incentive*** payment to employees who participated in a GWP, this triggered concerns that tended to mirror the responses of those who felt a GWP would diminish feelings about their employer. For example, one respondent said, “I would definitely be more inclined to take the test if they were offering a cash incentive. Money talks and times are hard, especially since COVID or whatever. So the extra money would definitely be a huge perk for me. But I’m with everybody else: Why are you offering a cash incentive? Why is it so important to you as a company that your employees take a genetic testing?” [White Female] In general, the addition of incentives seemed to raise suspicions and reduce feelings of trust for employers.

Respondents were also asked how they would feel about a GWP that generated genetic information about ***mental health risks***. This generated ambivalent feelings among many respondents. For example, one respondent shared: “When you have depression and anxiety, and I have a lot of family members who have this unfortunately, your production or your productivity and efficiency to do your job declines, because of your mental state. So if they were to use it to promote mental health or something along the lines of that, I would like that. But if it’s just to have information to see, “Oh, is this employee going to get depressed?” I think I wouldn’t like working for that employer, personally.” [Black Female] This comment highlights respondents’ perceptions that the same information about their genetic risks could be used both positive and negative ways. It also suggests that an employee’s trust in their employer could be impacted by their perceptions of the GWP it adopts.

### Effect of Genetic Wellness Program on Positive Feelings About Employer

When asked directly how their current employer offering a GWP would influence their feelings about that employer, respondents varied widely. Some reported that this would enhance their positive feelings, some said it would diminish them, and one respondent said it would make no difference. Respondents were not required to take a position either way. However, notably, those respondents who reported enhanced feelings were all White, and those respondents reporting diminished feelings were mostly Black. Representative quotes for each position are provided in Table 6.

**Table 6.** How GWPs Affect Positive Feelings About Employers.

Enhancing positive feelings about employer	offered as a service to employees, … to me it shows a company that’s really committed from a benefits and total rewards perspective to catering to the future of healthcare. [White Male]
	I think it’d be a great additional benefit. I love that it would kind of be outside of the insurance benefits that they would offer like an additional incentive for the company. [White female]
No effect on positive feelings about employer	I don’t think it’s going to make or break me staying at my job. It’s just one more added benefit. [White male]
Diminishing positive feelings about employer	My preferred way would be getting this through my primary care provider or a care provider that’s within my care group. Personally, they know my medical history. They would best know all of that information. Not guaranteed, but higher chance it would there would be privacy and that my data wouldn’t be moved around and things like that. [Black female]
	… sometimes people are worried that if the company is going to know that they’re going to fall sick or they have a tendency of falling sick, they may not be given a promotion, or a better responsible job. So, I think I can save all that if I know that my data is going to stay with me and my primary care [doctor]. [White male]
	But my first question was why would a company do this? What’s in it for them? So what’s the motivation on their side? We don’t want them to have our data. So why are they doing it? [Black male]
	I don’t want them knowing all my business … I may have something. If they know all this information, what if I didn’t? What if I was adopted? What if I don’t know who my father is? I’m just saying an example. How would they be able to get that information, or how would that help? Like I said, what if I got stuff I don’t want people to know? Are they going to send this to law enforcement? I don’t know. Connect me with a crime? I’m just saying. How would I be protected? I don’t feel like I would be protected. It seemed like they trying to know too much. I’m very extremely leery, not saying that I’m going to do anything. I just think they just trying to get too much information from me. [Black female]
	I think that actually brings up a good point, because with my family and my genetics, I have high blood pressure or I’m prone to high blood pressure and diabetes. So I think that brings up a good point. And then I also have cancer in my family. So, would they dismiss me because I have maybe a shorter lifespan than most other people? [Black female]
	What’s the benefit of your employer giving you this genetic wellness program instead of just giving you healthcare, and you go on and go to this genetic testing. What [she] was saying, how does this benefit the employer and not the employee? [Black female]
	What’s to stop the employer from buying back that same information. And then, like we talked about earlier about using it against you and saying, “I don’t want employees that are going to run up my insurance bill,” or “I don’t want to hire somebody and put time into them if they’re going to be dead in two years.” so just because they send the test results straight to me, doesn’t mean the employer’s not going to figure out a way to get a copy of the results. I guess it’s modern day paranoia. [White female]

For those who reported that a GWP would ***enhance positive feelings*** for their employer, their reasoning involved the potential for the GWP to demonstrate their employer’s commitment and caring about employees, and also the value they would place in having access to such an employee benefit.

For those who reported that a GWP would ***diminish positive feelings*** for their employer, reasons included: a preference for genetic testing to be conducted through their regular health care provider, concerns about information privacy, and concerns about employer misuse of information generated through the GWP. The latter concerns include various types of genetic discrimination in employee hiring, promotion, and dismissal. Although federal law prohibits discrimination against employees based on genetic information, there are questions about the scope and enforcement of that law.^12^

Finally, one Black respondent also specifically connected their diminished feelings for their employer due to offering a GWP to their African American identity: “I am African American. To me, I feel like I’m already going to be a target in the company. I don’t want my employer knowing. I feel like I want to keep something to myself. I would go on the outside if it’s something I want to do outside of the company to find out” [Black Female]. This respondent’s comment captures the possibility that many Black employees will respond to GWPs with extra concern, due to a generalized lack of trust in employer intentions and institutions.

## Discussion

This is one of the first studies to investigate how employees perceive GWPs, an important innovation in employer wellness programs. The vast majority of large employers already offer wellness programs, and this significant innovation could be offered in place or as a supplement to their existing programs. Employee attitudes may influence corporate adoption of GWP programs and employee participation where offered.

Findings indicate that nearly all respondents see potential benefits to GWP participation for themselves or their families. However, there are profound difference in perceptions of risks to GWP participation between Black and White/Asian respondents. These differences surfaced in three broad areas: privacy and discrimination risks; family impact risks; and feelings about the employer.

Perceived privacy and discrimination risks—which also exist in traditional wellness program biometric screenings and health assessments.^13-15^—appear magnified for GWPs. This may be due to the additional features of genetic data, which can reveal sensitive medical and non-medical attributes of individuals and their family members, and which are difficult or impossible to anonymize.^9,16-18^ Importantly, our research suggests that while these concerns were not a major barrier for White and Asian employees who were members of these focus groups, they were a major barrier for Black employees. Concerns about inaccurate information, misuse of information, genetic discrimination, and misuse by law enforcement were consistently raised by Black respondents. Some of these concerns were also explained as issues rooted in racial identity. African American distrust in medical institutions due to historical mistreatment has a well-documented impact on decisions to participate in biomedical research and seek medical care.^19-22^

Respondents also considered impacts on family members in their assessments of GWPs. Racial differences emerged here as well, particularly with concerns about how genetic information could be used by law enforcement in ways that harm family members. While these issues were raised by respondents from different races, they were expressed as participation barriers primarily by Black respondents, consistent with an awareness of historical racism in policing^23^ and racial implications of expanded police searching of DNA databases.^24^

Finally, Black respondents more often predicted that GWP adoption would diminish their positive feelings about their employer, whereas White and Asian respondents usually predicted the opposite effect. Black respondents explained this was in part due to concerns about employer genetic discrimination that persist despite existing some basic legal protections.^12^ This suggests GWPs could experience an even greater uptake-rate gap for Black employees than has been documented for traditional wellness programs.^25-28^ This finding is particularly salient in light of ongoing efforts by large employers to expand workforce diversity and improve racial inclusion.^29^

### Limitations and Future Research

This study’s findings are limited due to its reliance on a small sample of technology industry employees. While the present research surfaced and reported on differences between racial groups, we did not detect differences by gender. It is possible that such differences would have surfaced had we organized the focus groups along gender lines. Further, while we did not observe differences between White and Asian respondents, or between lower-income White respondents and other White respondents, future research is needed with larger samples of representative Black, White and Asian employees, as well as employees from other diverse demographic and industry backgrounds. Our focus group recruitment tool did not source many candidates of Asian or Latin American descent, even when we requested racial and ethnic diversity. Regarding Latin American candidates, this might reflect lower demographic representation in the high-technology industry where we focused our research recruitment. Based on our experience, future studies need to be designed in such a way as to enable recruiting and representation of these important demographic groups. In sum, future research with larger samples should examine differences by gender, more race/ethnicity categories, and intersectionality.

## Conclusion and Implications

The research reported here suggests an urgent need for attention to racial participation barriers for the new class of GWPs. There is recent recognition that other types of wellness programs could be better designed to address participation barriers for racial and ethnic minority employees and lower-income employees.^30,31^ If this opportunity were taken seriously for GWPs, they could play a positive role in helping employers achieve their racial equity goals and better align their broader workforce equity and wellbeing initiatives.

Taken together, our findings suggest the following specific implications for the design and regulation of GWPs:

• Addressing the concerns of Black employees appears critical for securing their participation in GWPs--and therefore also for avoiding unintentional furthering of employer-based racial disparities during GWP adoption.
• A first step toward addressing these concerns at the employer level could be to involve diverse employee stakeholder groups, such as employee resource groups or diversity councils, in GWP selection and/or design.^32^
• Given the nature of these concerns, however, addressing them fully will require that program participants retain controls over their genetic and health information, including how it gets shared and used. Such controls could constrain GWP business models that rely on monetizing participant data.^18^
• Crucially, addressing these concerns could also benefit all employees, and could increase trust and uptake of GWPs more broadly across the workforce.

**So What?**

**What is already known on this topic?**

Genetic Wellness Programs are a new form of employer wellness benefit that gives employees access to genetic testing. Only one published study reports employee attitudes about GWPs (and it was conducted using the employees of a genetic testing company).

**What does this article add?**

Our study provides important information about how employee perceive the benefits and risks of GWPs, and how a GWP would influence their feelings about their employer. In addition to overall results, we discovered distinct concerns and mistrust among Black employees, correlating with a much lower willingness to participate in GWPs.

**What are the implications for health promotion practice or research?**

Companies can expect most employees to support and likely participate in GWPs – but Black employees report concerns that represent barriers to participation. GWPs could contribute to health disparities unless redesigned to take these concerns into account.

## Appendix Screener Survey Text and Focus Group Discussion Guide

### Screener Survey

#### Focus Group Goals

The purpose of this study is to understand attitudes about workplace genetic wellness programs (GWPs). Specifically, the goals of the focus groups will be to gather qualitative data on:

• How employees view potential benefits and risks of GWPs
• Interest in participating in a GWP
• Attitudes about different ways these programs can be designed and governed
• How you would feel if your employer implemented a GWP

### Discussion Guide

#### 3:00pm Welcome and Ground Rules

• Hi everyone, thank you for joining. As we’re settling in, I want to offer you the option to rename yourself to whatever name you would like to go by - you can use your first name, initials, an alias, whatever feels comfortable to you. If you would like to rename yourself, take your mouse to the upper right corner of your own video tile, click the three dots, and select “Rename.”
• Thank you for agreeing to participate in this focus group about understanding your attitudes towards workplace genetic wellness programs.
• My name is Angel, and I am the facilitator for today’s conversation. I am here with my colleague Yvette, who will be taking notes. Yvette, could you say hello for a moment? Thanks, Yvette and I are on a research team working with Dr. Forrest Briscoe, Professor of Management at Penn State.
• Before we begin, I want to confirm that all of you had a chance to review the informed consent form. We shared through a link in the reminder email for this session. Can I please see a nod or shake of everyone’s head so I can see who’s had a chance to review it? Has anyone not had a chance to review it?
• If not all have seen: Okay, I’m going to take some time to review the content of the informed consent form. Essentially, the purpose of the research is to better understand public attitudes about genetic wellness programs, which will help inform organizations and policymakers about these attitudes and potentially contribute to the design of genetic wellness programs. Your participation in this research is confidential to the degree permitted by the technology used, and we will be recording this focus group session. If there are any publications or presentations that result from this research, there will be no personally identifiable information shared. You will be compensated $90 for your participation in this focus group, which is voluntary. You can stop at any time, and you do not have to answer any questions you do not want to answer.
• Great, thank you. Your nods and continued participation in this focus group are communicating consent to take part in this research. (As a reminder, your participation iis voluntary and you do not have to answer any questions you do not want to answer.) Today’s session will be recorded so that we don’t miss anything that you say. The recordings will be kept under password protection and deleted at the end of the study.

#### 3:05pm Ground Rules

• Now then, just to make sure all of our microphones are working, I’d like for everyone to speak up for just a moment. I’ll call on you one by one, and if you could please say hello and give your name you’re using for this session, that would be great. So for example, I would say, “Hi, I’m Angel.”
• Great, thank you. Onto some ground rules. It’s important that we hear from everyone and hear your honest thoughts, opinions, and experiences. There are no wrong answers. I ask that you please be respectful of each other.
• If you have something to add to the conversation, please feel free to jump in. However, ideally, we would like to have one person talking at any time. If you’d like, you can go ahead and raise your hand so we can see it on the screen, or you can also raise your virtual hand if you’d prefer. Down at the bottom of this Zoom video, there’s a “Reactions” button, and if you click on that, you’ll see a button for “Raise hand.” So you can use your real hand or your virtual hand if you’re trying to find a way in to the conversation. This will help us hear everyone’s thoughts and opinions.
• We have a lot to cover so we will try not to spend too much time on any one topic.
• Does anyone have any questions? Let’s get started.

#### 3:10pm Focus Group Discussion Questions

Today we will be asking you questions about your thoughts, feelings, and experiences when it comes to workplace genetic wellness programs. Genetic wellness programs are relatively new, and to make sure we all share some basic level of understanding, we have a little bit of information prepared for you about what they are, and the potential benefits and risks.

We’re going to start by sharing some text about the potential benefits of employer-sponsored genetic wellness programs, and talk a little bit about it. Then we’ll go over some text about the potential risks, and have some discussion on those.

We’ll first begin with the potential benefits, which will also give a little bit of background on what employer-sponsored genetic wellness programs are. I’ll read it out loud for everyone.

#### 3: 15pm Text #1

A genetic wellness program is an employee benefit that provides personalized information about your hereditary risks that can potentially help you, your family members, and others lead a healthier life. Genetic testing will provide information on:

• Cancer risk: A look at select genes to better guide a screening and prevention plan for common hereditary cancers including breast, ovarian, and colorectal.
• Heart disease risk: A look at select genes associated with genetic forms of heart disease, including hereditary high cholesterol.
• Medication response: Analysis of genes associated with how the body may process certain medications.
• Fun insights: How well do you digest dairy products? What is your earwax type? Are you likely to love or hate cilantro?
• Family members’ hereditary risks: Your results could identify relatives who could benefit from genetic testing, and help future generations know what to look out for.
• Topics under medical research: Your data could be used in research studies to help identify hereditary conditions and improve medical treatment.

How it works:

1. Claim your benefit and create an online account
2. Mail in your saliva sample kit
3. Receive your results online
4. Meet with a genetic counselor (if available)
5. Share the results with your doctor to create a screening and prevention plan (if applicable)

#### 3:20pm Questions about Text #1

I’m going to leave this text up for another 10 seconds or so, so that you can review it on your own briefly, but then I’ll be taking it down from the screen so that we can talk face to face.

(count down 10 seconds)

So that was a little background on employer-sponsored genetic wellness programs and the potential benefits they can offer. I’d like to open up the discussion now, and please don’t be shy about being first - my first question is:

1. What are your first thoughts and feelings in response to this information?
2. Do any of these benefits strike you as particularly valuable?
3. Are there other benefits that come to mind?

Great, thanks so much for your thoughts on the potential benefits of genetic wellness programs. We’re going to turn our discussion to potential risks, and I have some more text that I’m going to bring up onto the screen and read out loud.

#### 3:40pm Text #2

Employer-sponsored genetic wellness programs have potential risks, such as the following:

• Inaccurate understanding: The results may be easy to misinterpret or could be based on a misapplication of the science.
• A burden of knowledge: A genetic test can leave you with information you’d prefer not to have about your family or about your risk for an incurable disease.
• Lower-quality counseling services: While companies that serve employer-sponsored genetic wellness programs often offer genetic counseling, a company counselor will be less familiar with your medical history than a counselor your doctor refers you to.
• Loss of control over your data: Once data about your genes is shared, it can be sold to others for uses you may not be aware of.
• Potential discrimination: Though there are some laws in place to protect against using genetic information as a basis for discrimination, there are gaps in protection for different types of insurance and employees in small businesses.
• Use by law enforcement: Your data could potentially be used by law enforcement to identify suspects in crime scene investigations.

You can read more about these risks from the 2021 *Consumer Reports* article, “Read This Before You Buy a Genetic Testing Kit: At-home testing can offer an incomplete picture of disease risk, get ancestry wrong, and compromise privacy.**”**

#### 3:45pm Questions about Text #2

Again, I’m going to leave this text up for another 10 seconds or so, so that you can review it on your own briefly, but then I’ll be taking it down from the screen so that we can talk face to face. (count down 10 seconds)

So that was a little bit about potential risks of employer-sponsored genetic wellness programs. And now I’d like to hear from all of you -

1. What are your first thoughts and feelings in response to this information?
2. Do any of these risks strike you as particularly concerning?
3. Do you have other risks that you are concerned about?

Thanks so much for sharing your thoughts. I have some other more general questions I’d like to turn to now.

#### 4:05pm General Discussion Questions

1. Hearing about both potential benefits and risks of employer-sponsored genetic wellness programs, would you participate in such a program if it was adopted by your employer? Why or why not?
2. If an employer offered this program as an employee benefit, how would this affect your feelings about (or interest in) working for that company? Would it make you more or less likely to stay with that employer? Why?
3. If a genetic wellness program was available to you, are there options or features that would make you more interested in participating?”
4. There are other ways to access genetic testing services outside of your employer - most notably, through your doctor’s office or on your own. When thinking about these three ways to access genetic testing services, which would be the most preferable to you (if any)?

#### 4:25pm Closing

Thank you for sharing your thoughts and experiences today. We’re almost done with this focus group discussion, and before we finish, is there anything else you would like to add on any of the topics we discussed today?

Thank you for your time and participation in this focus group. Your comments will be very helpful to this research project. You’ll be receiving $90 through User Interviews as compensation for your time. It was a pleasure to talk with you all today. I hope you all have a great rest of your day.
